# Supplementary material for: Comprehensive transcriptome analysis of cochlear spiral ganglion neurons at multiple ages
Source: eLife. 2020 Jan 8;9:e50491. doi: 10.7554/eLife.50491 (PMC7299348; doi:10.7554/eLife.50491)
Supplement: Supplementary file 3. [file elife-50491-supp3.docx]

**RNA in situ probe sequences**

**1）Elavl4**

TCAGGATTTGTGGGCTTTGTTGGTTTTAAAGGAAACTTGCAACACTCTGTCTCCCAGGCGATAGCCATTGAGGCTGGCGATGGCCATGGCTGCCTCATCGTAGTTGGTCATGGTGACGAAGCCGAATCCCTTGCACTTGTTGGTGTTGAAGTCACGGATGACCTTGACGTTGTTCACTGCGCCAAAGGGGCCAAAGAGCTGCCAGAGGACACTCTCATCAGAATCAGGGGACAGGTTATAGACGAAGATGCACCAGCCTGTCCCTGTGTGACCAGGGATGTTCATTCCCACAAGACTTGTCATCCCGTCAATGGTGATTGGGGAGAAC

**2）Esrrg**

ATGTTTAGTCAGGGATTTGCCAATTTTTTAAAACTTATCACAGCTATCAATTTCAAGTTATTGACCATCCGAATGAATTGCTAATGATGATTTACCTAGAGTCACACTACAGTCACTTTCTCTACTGAGAACCACAATCTACGATATATTCCCATATAGCTAATGATAGATACACAGTATTCCTAGCACAGGGCTGAGACACCCTCAGTCCAAGGTATTTTGCTTAACGCACACAAAGTAGCAATAGTTTTGGAAGCAAGTCCGCACCAAAAAGGTTTGCAACAACACAGAGCGAGTTAATAACAACATCTGAGAGATGATCAGGTGGAAGCCTTATATACAGCATTTAAAAATTAGACTTATCTATATTCTTGAATATTCAAAGTTTTATGTCTAAGCTATACATTGGTATTCTATAATAAACCATTAGAGAAATTATCCCTTTTTTGAAAATATTATTATGATTTTGATGTGTGTCTGATCGTAATGAATACTGTTTTGGAGTTTCAATCTCTTAGTTATTACTGGTAAGGAGTAGAATATACTACTGTAGTCTCCTGAGGAAGTATACATCCAATACTGTCTCTAATTCTACCCTGTGTCTAAAAGCACACACCACAAGAATCCAAACACCACGAGAACAAGACGACAGTTTCTGAATGC

**3) Scrt1**

AACATAAGGCCCAAGTCAAAGGCAGAGCCTGACTACACCCAGCGTATTCTAGGACTGAGGTGGGTAGGAGAGGCCATAGGGGCCCAGGGATTCAGCCCTCTGCATCTGCCTTCCACCCTGGCCTACCTATGCGTATTGGGCCCTGTCACTGATGGGCACCTCCAGAAGGTTGCCACATCTCTGACGGATTCAGGAATGGGCCTGGCATATCGTAGGTACCCAATGATGGGTCGGTGAGTGATGAAGGACTGACAAGAGAGGACTTGGGTGATACCATTCCATCCCAGCCTCTGGAACCATGACGCCTGCCGGGCTCTGCCCTGGGGTAGGGTATGGGGAAAGAAAGTCTGCGTGGCCGGCCCCGCGCAGCATCCCTGATCTAGGGGCCTATGGGATGGGTAGGGCAGGGGATACCTGATTCTCAGTAACTCTAGAGGCTGCGGCAGCTTCACATGCAGTGCGCATTATTGCTCTATAGTCTGCATCGGA

**4) Scrt2**

TCCTAAGGAGTTGAGGCTCTGATAAGGGTGGGCTTCTCCCCAGACCCATAAGGGGAGGAGCAGCCAGCGACGGACGGCCTTGCTGACTCTACCCTTTGACTTCCCAGTCCAGCTGGGGAAGCCAGACAGTCCCTGGGTGAAGCCAAGAGATTAAATAGAATAATAAATAGATAAATAAATAAATACACAAATAAATAGCGGAAGGGGCCCTGCCGGACGAGGGGATGTGTCCATGGGTGAGGTGCTCCAGGCTGTTCTCTAGCAAAGGCAGAGGCGAACCTGTAGTCCTGGGGCGAGGGAAAGTGCTCAGATAGTCTGGGAAATCGCAGCTCCAGGCCTGAGGCCTAGACAGGAACTTTCCTGGGGCAAGAGGGGTCACCTGAGAGGGCAGAGCCTTGGGCTGCCTCCCAAGTCTGTTCCCAGCTAGGAAAACTGAGGACTGGGAGCTACTAGTGGGAAGATGGAGCTGGAGAGAGCAGGATGTCTGATGAGATCCGGGAGGACTGATAATGTCAGGTGGAATCGTGTGTGTAAGAGGCTGGACCTAAGGCCCAAGCATGGAGGGGCTCAGGAATGGACAGGCAGAGGACTCCAAGAGTGGTGAAGATTAGATTACGGTATGGGGACACAC

**5) Stmn3**

TAAGAGAGCCCCAGCCACACCTGGCCTAGTGTCTGAATAGGGGACCATATCTCCTGCCTAAGGAGCTCCTTCAGGAAGAGCCCAAGGTCAGACACGTTACAAGGACTGGACAGTTCAGAGTGTGGGAAAAAAGCAGGAAGCTGAGGGGTACAGGGGAGCAGATAGGAGCAGGCACAAAATTCGCATCTAGACAGAATTGAAAACAAAAACAAAACCCGAAAACGTTCTTGTCGCCGCTGGGTCCAATACTCCTTAGCCAGACATTTCCTCCCGCTGCTCCTTGTTCCTGCGCACCTCAGCAGCGTGCAGCTCCTTCTCGCGCAGCCGCTCGCGCAGCGCTGCCAAGTGCGCCTCGCGGATCTCCTTGCTCAG

**6) Shox2**

CTCTATCCAGACTTCCCCAAACCCGCTCCTACAAAGCCGAATTTTAGTCCCAAGGGCGGGTGGGCAGGAGCCCCTGGGCTGGTGGCCCTCGTGGGATCCCGGGTCCAGCGCAGAGTCCCGCCCTGGGGATCCAGGGTGCAGAAAGAAAAGGCGGCAGTCAGCGGGGAAGGGACAGGCCTGGGCGACGGTCAGAAGCGGGTGCGGGAGAAGCCGGTCGCGGAGGGCGTGCAGGATCGCGCCGCTTAGGAGGCTCCACCGACCGTGGCGCTGGCGCCGGCGTCACAGACCCAGGGCGGCCGCGTGCTTTTTAGCTTTCAGTC

**7) Gabbr2**

GTCGTAGATAAGGCTCGAGATCCGTGCTGAGTCCCTCTCTGCCTGAGCGCCATTCCAGCAGTCTGGAGAGGCAGCATGGTGCCCAGCTTCTCGCCGCCGTCAGCGTGCCAGTGGCAGGCGCCTTGGCCCAGCATGGTGCTGTCCCGGTGAGGCCCGGCCCCAGGCCTCCCTGCCCTACAGGCCCGAGACCATGACTCGGAAGGAGGGTGGTACGTGTCTGTGGCGAGGGCTGGCTGTAGGGCTGACACAGGGGCTGACGCAGCTGGCGTCCACGCCTCCAATGGACGGGAGGTAGGCGTGGTGGAGGATGGGGAGCTGGAGGGACAGCCGGCGCTGGATGTGCTCTGGGGAGTTGATGTCCTCTATGGGGTCTTTGCATGTTCTTGAGGGCTCGGTCGTGTTCCACTGGAGCTGAGGGTTTTGATCGAGGTGATTTTTTAGAATGGCCTTTCCTCCATCTGTGCTCTCTGTGAAGTTGCCCAAGCTGAGGATGTCGTTGAGCTCTTGGTAGTGATTCTGTTTGATGTATGTGGTCTTCTCTGGTGTGTCTTGTAGCTGCATGGTGACTTCTTCCAAGTCTTTGTCCAGCTCTGTGATCTTCATTCGTAGGCGGTGGTTTTCTGACTGCAGTCCCTCCAGGCGTGACGTGCTCGCCTGGTTCACGCTGGTGACTGAGGTGGAGGTCTTCGAATCTTCTTTCTTCTGGTTCTGTGTGAACTGGAATCGCCTGTTCTGAGTGGCTGCGTCAGGGTTTGTCCTCAGAGTGATGAGCTTTGGCACAAACACCAGGCAGAGAGTGATGGTGCTACAGAAGATGATGACCAGGGCCACGATGCAGAACTGCACGTTGGGCTGGTCACGTGTCAGGAAGGAGACGGCGGCCCCGATGATGCACATGATCCCCACATTGTACACACTCATCCCAATGTACTTGCTGTC

**8) Celf4**

GTCCTGCATACTGCTGCACTCCAGCGTAGGCCTGCTGCAGGGGGTCGGCTGCGGTGGGGCTCTGTGCTGGGTAGGGGTGAATGCCATTGGCAAACACAGCTTCCGCAGCAGGCTGCCCATTGGCCTGAGGGGGGAGGCCCGTGAAGCCGTTCACCCCAATGGGGGATGGGATGCTAGGCACAGCTGGTGCAGTGATGCCTGGAGGGGTGCTGCCACCTGAGGTTGGGGTCATAGGTGCGGCTGCCAGGCCATTCATGTTGAGGGCCGCCATCTGCTGCATTTGGGCGGCAGCGAAGGCAGCCATGGGATTCAGGTAGCCTCCTTGCGCGACCGATGCCATGAGGGCTGCTTGCTGCTGCATCAGTGCCTGAGCATAGGCGCCATAGGCTCCGAAGGGGATGGCCATGGGGTTGAACATGCCCATCTGGCCAGCCATCTGCTGCATCCGTCGCATTGTGCGCTCCTTGTCAGTGTCTGCAAACTTGACCACCAGGCTGGAGGAGGCTCCAGGCATGGTCTGGCTGCCATGTAGAGCGTTGATGGCGGCTTGTGCCTCGGCATGGGAGGAGTACTTCACAAAGGCGCACC
